# Evaluation of the expression level of microRNA-21, microRNA-15a, microRNA-372 in human follicular fluid stem cells-derived oocyte-like cells (OLCs)

**DOI:** 10.5935/1518-0557.20240019

**Published:** 2024

**Authors:** Ghasem Saki, Kousar Shahrooie, Mahin Taheri Moghadam, Ali Reza Eftekhari Moghadam, Roshan Nikbakht

**Affiliations:** 1 Cellular and Molecular Research Center, Ahvaz Jundishapur University of Medical Sciences, Ahvaz, Iran; 2 Department of Anatomical Sciences, Faculty of Medicine, Ahvaz Jundishapur University of Medical Sciences, Ahvaz, Iran; 3 Department of Anatomical Sciences, Faculty of Medicine, Baqiyatallah University of Medical Sciences, Tehran, Iran; 4 Fertility, Infertility and Perinatology Center, Imam Khomeini Hospital, Ahvaz Jundishapur University of Medical Sciences, Ahvaz, Iran

**Keywords:** oocyte-like cells, microRNA-21, microRNA-15a, microRNA-372, infertility, *in vitro* fertilization

## Abstract

**Objective:**

Today, researchers have succeeded in achieving oocyte-like cells through the in vitro differentiation of stem cells. MicroRNAs are key regulators of oocyte development. In this study we decided to evaluate the expression pattern of microRNA-21, microRNA-15a, and microRNA-372 in oocyte-like cells, to determine the maturation stage of oocyte-like cells.

**Methods:**

Human follicular fluid samples were collected and centrifuged, and their cells were divided into 3 groups; day 7 as control group, days 14 and 21. During this period, the cells were evaluated for their morphological appearance and viability by inverted microscopy. RNA isolation was performed and cDNA was reversely transcribed by specific stem-loop RT primers. Real-time RT-PCR was used to detect microRNA expression.

**Results:**

The relative expression of microRNA-21 and microRNA-15a on day 21 was significantly down-regulated compared to the control group (day 7), but microRNA-372 did not show a significant difference. Also, on day 14 compared to the control group (day 7), microRNA-21 did not show a significant difference; but microRNA-15a and microRNA-372 were significantly down-regulated. MicroRNA-21 and microRNA-15a on day 21 compared to day 14 revealed down-regulated levels, but microRNA-372 revealed up-regulated levels.

**Conclusions:**

Our results showed significant decreases in the expression of microRNA-21 and microRNA-15a in oocyte-like cells, as well as in oocytes, which may lead to cytoplasmic maturation, germinal vesicle break down and the completion of meiosis І. In addition, down-regulation expression of microRNA-372 maybe a confirmation that mesenchymal stem cells have differentiated into germ cells, and these cells were differentiated into oocyte-like cells.

## INTRODUCTION

Infertility is one of the main problems couples may face, and it is increasing despite various treatments, at an overall average of 10% in the world (Direkvand-Moghadam *et al*., 2016). The methods used to treat infertility are called Assisted Reproductive Technology, or ART (Direkvand-Moghadam *et al*., 2014). In this method, the ovarian follicle is removed from the ovary, and in the laboratory, after separating the oocyte, the follicular fluid is discarded (Astbury *et al.,* 2020). Follicular fluid is an essential component of the ovarian follicle and contains hormones, enzymes, electrolytes and antioxidants for oocyte maturation. Also, the follicular fluid has several types of cells (Taheri Moghadam *et al*., 2021; Rungsiwiwut *et al*., 2021).

It has been shown in studies that ovarian follicular fluid-derived cells are stem cells; they also express stem cell markers CD45, CD34, CD90, CD133, SSEA4, OCT4, and NANOG (Rungsiwiwut *et al*., 2021; Koliakos *et al.,* 2019). Furthermore, researchers demonstrated that follicular fluid stem cells spontaneously differentiate into oocyte-like cells (OLCs) in vitro and express oocyte-specific markers such as Zp2 and Zp3 (Taheri Moghadam *et al*., 2021; Virant-Klun *et al*., 2008). According to these data, these cells can be used to investigate the processes of oocyte growth and differentiation during oogenesis *in vitro*. Oogenesis is controlled by several molecular processes. One of the effective molecules in these processes is microRNAs; which are small, single-stranded, noncoding RNA molecules with 18-24 nucleotides. MicroRNAs bind target mRNA by the RNA-induced silencing complex (RISC) to prevent mRNA translation and protein production; therefore, they play an important role in the posttranscriptional regulation of gene expression in human reproduction (Li *et al*., 2015; Wahid *et al*., 2010). The expression of microRNAs in oocyte has been reported in various mammalian species, including human (Xu *et al*., 2011), bovine (Abd El Naby *et al.,* 2013), mice (Yao *et al*., 2014), and pig (Hale *et al*., 2020), and they can control the expression of genes related to oogenesis, including; oocyte development, cytoplasmic maturation (Jenabi *et al*., 2023), meiotic proteins formation (Liu *et al*., 2010), and cause the progression of the oocyte from the germinal vesicle stage to meiosis ІІ (Wright *et al*., 2016).

Up to now, studies investigated the expression pattern of microRNAs in oocyte in vitro maturation (IVM), but there is no report of microRNAs expression in OLCs in vitro. For this reason, to determine the maturation stage of OLCs, we decided to evaluate the expression levels of microRNA-21, microRNA-15a and microRNA-372 in these cells in vitro, so that we can use OLCs to treat female infertility in the future.

## METHODS AND MATERIALS

### Collection of normal follicular fluid samples

This study was approved by the IRCCS Bioethics Committee (Protocol number IR.AJUMS.MEDICINE.REC.1400.0497). Patients gave their written informed consent on oocyte retrieval day and were informed about FF sample collection. The inclusion criteria included women with no disease and who were referred to the infertility center of Imam Khomeini Hospital in Ahvaz for IVF due to male infertility of their husbands. Exclusion criteria included women with polycystic ovary syndrome (PCOs), premature ovarian failure (POF), thyroid gland disorders, blood pressure disorders, endometriosis and autoimmune diseases. The number of samples included in the study was 12.

### Isolation and culture of follicular cells

First, 40-50 ml of human ovarian follicular fluids was collected from each woman who was undergoing IVF therapy during oocyte retrieval through an ultrasound-guided aspiration needle (Lai *et al*., 2015; Riva *et al*., 2014). After identifying cumulus-oocyte complexes (COCs) by the first operator for IVF purposes, when no more oocytes were observed by the second operator, follicular fluid (FF) was pooled into a conical 50 ml falcon tube containing two drops of heparin (10-30 IU/ml). We used hypo-osmotic lysis to enrich the follicular cells and the elimination of red blood cells from FF (Lobb & Younglai, 2006). In this technique, follicular aspirates were centrifuged at 800g for 30 min. Then, aspirating supernatant was collected and the cell slurry was transferred into a 15 ml falcon tube. In the next step, 9.0 ml of the sterile distilled water was poured into the cell slurry, and the tube was blended. After 20s, 1.0 ml of 10× concentrated phosphate-buffered saline (PBS, pH = 7.2) was added and mixed in the tube. The tubes were centrifuged at 400g for 10 min again. Finally, the cell pellet was resuspended in 0.5 ml of Dulbecco’s modified Eagle’s medium (DMEM; Sigma-Aldrich). Cell counting and viability test were performed on aliquots in 0.2% trypan blue by a hemocytometer (Lobb & Younglai, 2006). FF-aspirated cells were seeded onto four-well plates (BD Biosciences) at 1 × 10^5^ cells concentration. The growing cells were separated in a DMEM supplemented with 10% fetal bovine serum (FBS; Gibco-BRL) and 1% penicillin/streptomycin (Gibco). Non-adherent cells were thrown away after 48h; the cultures were kept at 37°C in a 5% CO_2_ humidified atmosphere and monitored daily. The culture medium was replaced every 2 days. The samples were divided into three groups: day 7, day 14 and day 21. The cells were monitored daily through an inverted microscope (Nikon, Japan) for morphology assessment. Every two days, the cell culture medium was changed. The size of cells was measured by software: Analysis LS Research 3.2 (Olympus company) (Taheri Moghadam *et al*., 2021).

### RNA extraction and cDNA synthesis

The extraction of total RNA was conducted from 4-610^6^ using the SinaPure RNA Mini Kit (SinaClon). According to the manufacturer′s instruction. First, lysis buffer and precipitation solution were added into each sample. After that, all the samples were centrifuged and they were washed twice with wash buffer, centrifugation was done again. Finally, RNA samples were eluted in a 50 ml of RNase-free water. The quality of the extracted RNA sample was determined by the NanoDrop™ 2000c spectrophotometer at an optical density of 260 to 280, which is the best case, close to 2, and it was 1.98 in the extracted samples. Later, 500ng of total RNAs underwent reverse-transcription into complementary DNA using the cDNA synthesis kit (SinaClon), which include dNTP, gene-specific primer, M-MuLV Reverse Transcriptase, 5x Buffer M-MuLV and RNase inhibitor. Then, all the samples were left for 50 minutes at 50°C, ending in 15 minute-cycles at 70°C in an eppendorf Master Cycler. Finally, all the samples were stored at -20°C prior to RNA purification (Zhang *et al*., 2007).

### Quantitative RT-PCR

The real-time method was used to quantitatively check the expression of microRNAs. The volume of the real-time mix for each reaction included: 10µl Real Qplus 2x Mastermix Green (Ampliqon, Denmark), 2 µl cDNA, 1µl primer reverse, 1µl primer forward, 6µl distilled water and each sample was analyzed in duplicate. The temperature program of the real-time device (ABI 1500 PCR) in each cycle included: initial denaturation 95°C for 15 minutes, denaturation 95◦C for 20 seconds, annealing 60°C for 20 seconds, extension 72°C for 20 seconds. There were 40 cycles and the expression of U6 was used as a housekeeping control gene (Naji *et al*., 2018). The sequence of the primers used in this research is according to Table 1.

**Table 1:** Sequence of specific primers used in RT-PCR analysis.

Primer	Sequence (5′→3′ )
miRNA-21 forward	TAGCTTATCAGACTGATGT
miRNA-15a forward	TAGCAGCACATAATGGTTT
miRNA-372 forward	CCTCAAATGTGGAGCACTAT
Reverse qPCR	CGAGGAAGAAGACGGAAGAAT
U6 RT	CGCTTCACGAATTTGCGTGTCA
U6 forward	GCTTCGGCAGCACATATACTAAAAT
U6 reverse	CGCTTCACGAATTTGCGTGTCAT

### Statistical analysis

The formula 2^-∆∆Ct^ was used to calculate the relative expression of genes. Statistical analysis was done by using the independent-sample T-test and one-way ANOVA. The Graph Pad Prism6 (Graph Pad Software Inc., San Diego CA, USA) was used to draw the graphs. *P* value ≤ 0.05 was considered statistically significant (Xu *et al*., 2011).

## RESULTS

### Morphology of normal follicular fluid cells in vitro

**Figure 1:**
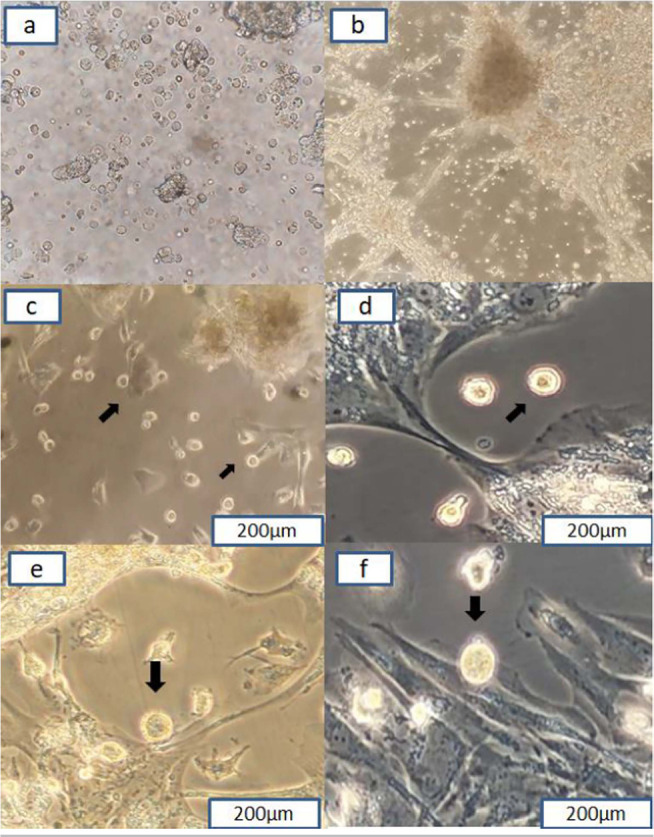
Cells derived from follicular fluid on the first day of culture (a). On day 3 of culture and cell colony formation and appearance of fibroblast-like cells (b). On day7, OLCs next to epithelial and fibroblast-like cells(c). On day 14 of culture (d). On day 17 of culture (e). On day 21 of culture, OLCs (f). Magnification 20x; arrows point to the OLCs.

The isolated cells on the first day of cultivation had a round morphology (Figure 1a). After growing the adherent small mesenchymal cells, they attached to the bottom of the 4-well plate and formed a cell colony over time, and fibroblast-like cells appeared in between these cell colonies (Figure 1b). On day 7 of the culture, oocyte-like cells appeared in the medium; they had a size of 20µm and appeared next to epithelial cells and fibroblast-like cells in the environment (Figure 1c). Gradually, over time, OLCs increased in size, so that, on day 14 of the culture, they reached a size of about 25 µm (Figure 1d), on day 17 of culture, the size of the cells was 27 µm, and finally on day 21, they became larger and reached the size of 33 µm, and they were located next to fibroblast-like cells and the cell colonies (Figure 1e, f).

### Real-time PCR results of microRNA gene expression analysis

#### Expression of microRNA-21

**Figure 2:**
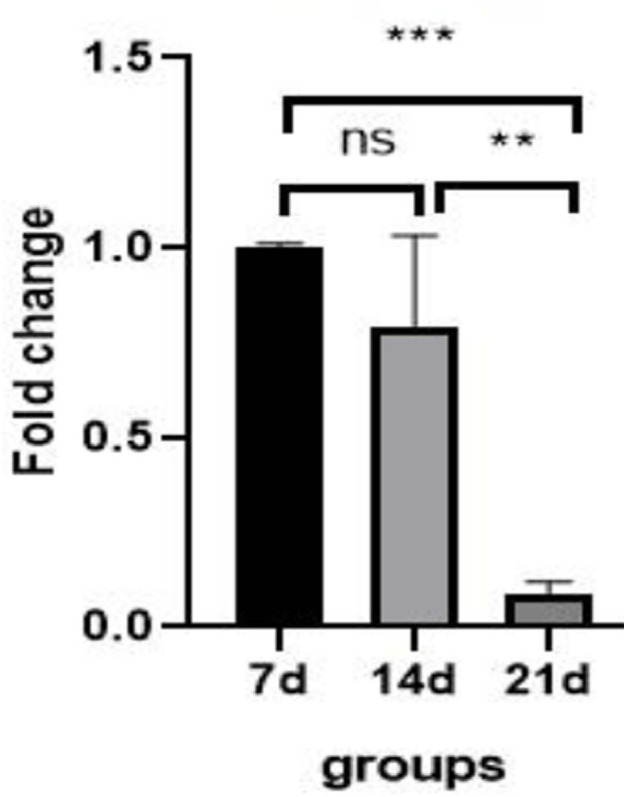
Expression of miRNA-21 gene in OLCs in control group (day 7) and study groups (day 14 and day 21). ***: *p*=0.0005, **: *p*=0.0022, ns = not significant.

As shown in Figure 2, the expression of microRNA-21 on day 21 was significantly down-regulated compared to that of the control group (day 7) (*p*=0.0005, 0.1-fold), but on day 14 compared to the control group, there was no significant difference (*p*=0.24). Also, expression of microRNA-21 on day 21 compared to day 14 was significantly down-regulated (*p*=0.0022, ~ 0.1-fold).

#### Expression of microRNA-15a

**Figure 3:**
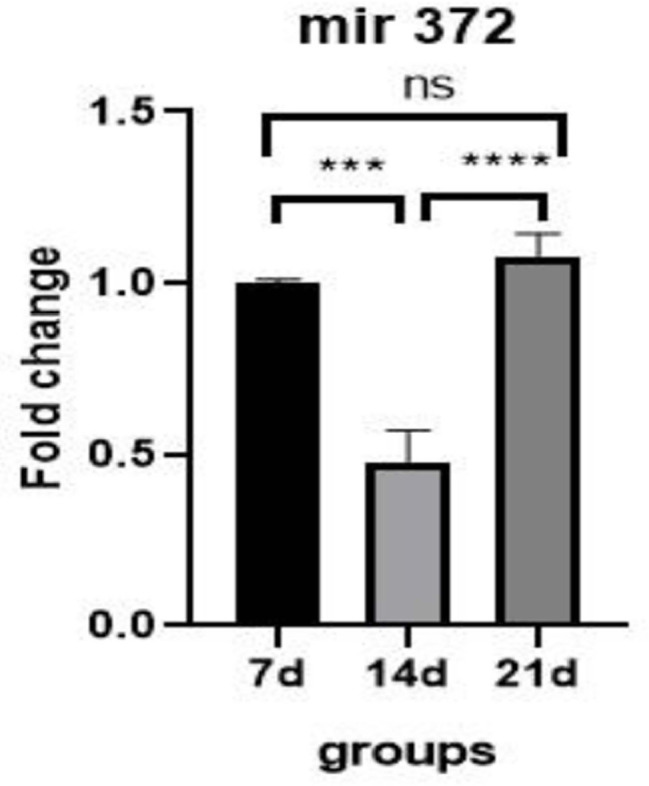
Expression miRNA-15a gene in OLCs in control groups (day 7) and study groups (day 14 and day 21). **: *p*=0.0019, ****: *p*=0.0001.

As shown in Figure 3, the expression of microRNA-15a on day 14 was significantly down-regulated compared to the control group (*p*=0.0001, 0.25-fold). Also on day 21, compared to the control group, it was significantly down-regulated (*p*=0.0001, 0.1-fold). On day 21 compared to day 14, the expression of microRNA-15a was significantly down-regulated (*p*=0.0019, 0.4-fold).

#### Expression of microRNA-372

**Figure 4:**
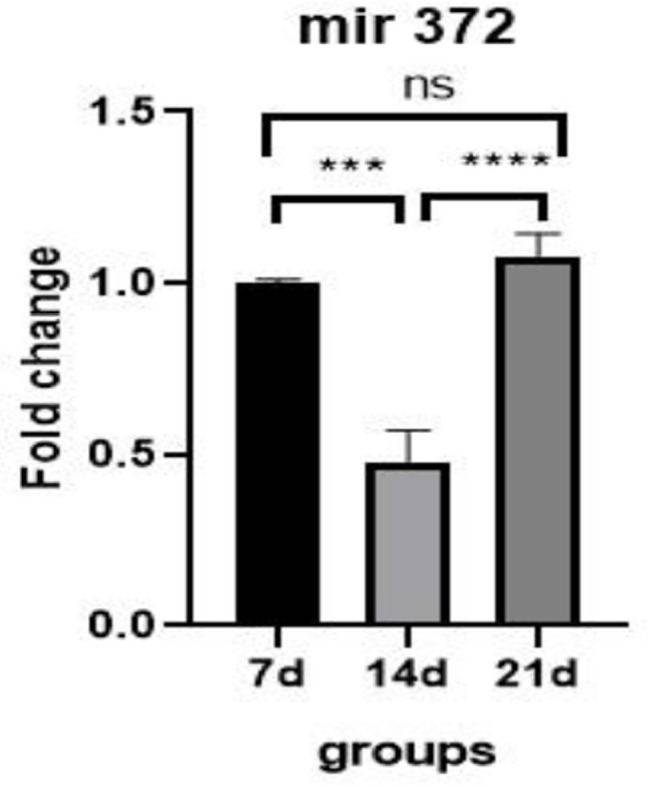
Expression of miRNA-372 gene in OLCs in control groups (day 7) and study groups (day 14 and day 21). ***: *p*=0.0002, ****: *p*=0.0001, ns=not significant.

As shown in Figure 4, the expression of microRNA-372 on day 14 was significantly down-regulated compared to the control group (*p*=0.0002, 0.46-fold), but on day 21 compared to the control group did not show a significant difference (*p*=0.42). On day 21 compared to day 14, the expression of microRNA-372 was significantly up-regulated (*p*=0.0001, 2-fold).

## DISCUSSION

The process of oocyte maturation includes dynamic epigenic mechanisms and bidirectional interaction between the oocyte and surrounding somatic cells. As a result, it leads to progress in the molecular processes related to folliculogenesis and eventually, development of the oocyte from meiosis І to meiosis ІІ. One of the key regulators of these processes are microRNAs, which play an important role in fertility. According to the studies, microRNAs had different expression patterns in different stages of oocyte development, including meiosis І, meiosis ІІ and germinal vesicle break down (GVBD) stage. In this study, for the first time, we investigated the expression level of microRNAs in oocyte-like cells in order to find out in which differentiation stage these cells were. Maybe these cells can be used as substitutes for oocytes in the treatment of women whose infertility problem is due to lack of oocytes or ineffective oocytes (Maalouf *et al*., 2016; Hossain *et al*., 2012; Gilchrist *et al*., 2016)

In this study, cells isolated from follicular fluid on the first day had a round shape, then, cell colonies were gradually formed, and a heterogeneous population of epithelial-like fibroblast-granulosa cells appeared in the medium on day 7. According to previous studies, these cells have the ability to express mesenchymal stem cell markers, including OCT4, NANOG, and SSEA4, so they can differentiate into different cell lines, including cartilage cells, adipose cells, and bone cells. Also, they are able to differentiate into germ cells and express DDX4, which is a germ cell marker. Therefore, these cells are mesenchymal stem cells (Rungsiwiwut *et al*., 2021; Koliakos *et al*., 2019).

One of the characteristics of oocyte maturation is the presence of zona pellucida (ZP) glycoprotein around the cell, which is a type of extracellular matrix and consists of 4 types. This protein has an important role in the development of the oocyte during the process of oogenesis, the interaction between sperm and egg, and the development of the embryo during fertility in women (Wassarman & Litscher, 2022). In the present study, on day 7, the stem cells were differentiated into round and small cells, which gradually increased in size over time. Previous studies have indicated that these cells are able to express oocyte surface proteins, which are ZP2 and ZP3 (Taheri Moghadam *et al*., 2021; Azandeh *et al*., 2022). As a result, they are oocyte-like cells.

In the present study, OLCs were 20µm in size on day 7. Gradually, their size increased so that they were 25µm on day 14 and 33µm on day 21. These results are also consistent with Lai’s study. Lai *et al.* (2015) showed that human follicular fluid epithelial cells formed cell colonies in the culture medium, and after some time they differentiated into oocyte-like cells, and these cells during the culture period from the second week to the fourth week had different sizes.

In our study, for the first time, we investigated the expression of microRNA-15a, microRNA-21, and microRNA-372 in oocyte-like cells derived from normal human follicular fluid stem cells. In this context, studies show that microRNAs play an important role in regulating cellular processes, including cell differentiation, apoptosis, and cell division (Trohatou *et al*., 2014). One of the microRNAs investigated in this study was microRNA-21. MicroRNA-21 has a regulatory role in many biological activities of the cell; therefore, it is considered a biomarker for the diagnosis of diseases including cancer and heart diseases (Jenabi *et al*., 2023; Jenike & Halushka, 2021). MicroRNA-21 also plays a vital role in the differentiation of mesenchymal stem cells, and its inhibition causes a decrease in the differentiation of stem cells (Sekar *et al*., 2015). Dehghan *et al*. (2021) conducted a study with the aim of evaluating the effect of microRNA-21 on the development process of mouse oocytes and their blastocysts in *in vitro* maturation (IVM). They showed that the increase in the expression of microRNA-21 in cumulus cells decreased the progress of mitosis, decreased the amount of Glutathione-S-transferase (GSH), and the expression of BMPR2 and PTX3 genes in oocytes. On the other hand, in oocytes, decreasing the expression of microRNA-21 increased cytoplasmic maturation, but it had no effect on the maturation of the oocyte nucleus or its progress from the germinal vesicle stage to meiosis ІІ.

Also, in this study, we investigated the relationship between the expression level of microRNA-21 and the OCT4 factor. They observed that the expression level of the OCT4 factor was significantly reduced in the blastocysts, which induced the microRNA-21 silencing factor (Dehghan *et al*., 2021). As we know, OCT4 is a pluripotency factor in mesenchymal stem cells. In the study of Azandeh *et al*. (2022), the expression level of this factor during the culture of normal human follicular fluid stem cells has gradually decreased, and this indicated the differentiation of stem cells into oocyte-like cells and the reduction of stem cells in the culture medium. In our study there was a significant down-regulation in microRNA-21 expression during the culture in the second and third weeks. These changes are consistent with the study of Dehghan *et al*. (2021), and similar to the expression pattern of microRNA-21 in mouse oocytes. It is also possible that the decrease in the expression of microRNA-21 and OCT4 factor in the culture medium indicates the differentiation of stem cells into oocyte-like cells, which according to Dehghan’s study (Dehghan *et al*., 2021), maybe oocyte-like cells are like oocyte in the cytoplasmic maturation stage.

Another microRNA studied was microRNA-15a, which belongs to the microRNA-15/16 cluster. This class is known as a tumor suppressor and works by targeting the BCL2, MCL1, CCND1, and WNT3A genes. As a result, by affecting the cell cycle of cancer cells, they reduce cell proliferation, promote apoptosis and suppress tumors (Aqeilan *et al.,* 2010). In 2011, Xu *et al*. investigated microRNAs expression during human oocytes meiosis *in vitro*. They showed that the expression level of microRNA-15a was significantly down-regulated during the development process of human oocytes from the germinal vesicle stage to meiosis ІІ (Xu *et al*., 2011). In our study, on days 14 and 21, compared to the day 7, the expression level of microRNA-15a was significantly down-regulated. These changes are consistent with the study of Xu *et al*., and similar to the expression pattern of microRNA-15a in human oocytes. As a result, according to the study by Xu *et al*. (2011), maybe oocyte-like cells are in GVBD and completion of meiosis I.

MicroRNA-372 was another microRNA investigated in this study, which has an anti-oncogenic role in tumors involving germ cells, ovarian cancer, gastric adenocarcinoma, and liver cells. MicroRNA-372 plays its role through proteins participating in apoptosis and the cell cycle, thus reducing cell proliferation and promoting apoptosis (Guan *et al*., 2017). Tran *et al*. (2016) showed that microRNA-372 increases during the differentiation of stem cells into primitive germ cells. This researcher reported that microRNA-372 can increase primordial germ cells by suppressing multiple cell cycle pathways as well as epigenetic processes (Tran *et al*., 2016). On the other hand, Rungsiwiwut *et al*. (2021) showed that follicular fluid stem cells can differentiate into germ cells and express the DDX4 marker. In our study, the expression level of microRNA-372 was significantly up-regulated on day 21 compared to day 14, which may indicate that in the second week (day 14), follicular fluid stem cells are more differentiated into germ cells. But the expression level of microRNA-372 was significantly down-regulated on day 14 compared to day 7; maybe it was because the stem cells were more differentiated into OLCs during the first and second weeks.

The results of the present study showed that, the expression pattern of microRNA-21 and microRNA-15a in oocyte-like cells is similar to the expression pattern of these microRNAs in the oocyte. As a result, it can be said that the OLCs in the present study have probably progressed to the stage of GVBD and the completion of meiosis І; and cytoplasmic maturation has taken place simultaneously with these changes. Also, the significant reduction of microRNA-372 was a confirmation that mesenchymal stem cells (MSCs) have differentiated into germ cells. Then these germ cells were differentiated into OLCs, but confirmation of this issue requires further investigations.
